# Gene expression-based clinical predictions in lung adenocarcinoma

**DOI:** 10.18632/aging.103721

**Published:** 2020-08-05

**Authors:** Yanlu Xiong, Jie Lei, Jinbo Zhao, Yangbo Feng, Tianyun Qiao, Yongsheng Zhou, Tao Jiang, Yong Han

**Affiliations:** 1 Department of Thoracic Surgery, Tangdu Hospital, Fourth Military Medical University, Xi'an, China; 2 Department of Thoracic Surgery, Air Force Medical Center, PLA, Beijing, China

**Keywords:** lung adenocarcinoma, gene expression, models, survival, recurrence

## Abstract

Mining disease-related genes contributes momentously to handling lung adenocarcinoma (LUAD). But genetic complexity and tumor heterogeneity severely get in the way. Fortunately, new light has been shed by dramatic progress of bioinformatic technology in the past decades. In this research, we investigated relationships between gene expression and clinical features of LUAD via integrative bioinformatic analysis. First, we applied limma and DESeq2 packages to analyze differentially expressed genes (DEGs) of LUAD from GEO database and TCGA project (tumor tissues versus normal tissues), and acquired 180 down-regulated DEGs and 52 up-regulated DEGs. Then, we investigated genetic and biological assignment of theses DEGs by Bioconductor packages and STRING database. We found these DEGs were distributed dispersedly among chromosomes, enriched observably in extracellular matrix-related processes, and weighted hierarchically in interaction network. Finally, we established DEGs-based statistical models for evaluating TNM stage and survival status of LUAD. And these models (logistic regression models for TNM parameter and Cox regression models for survival probability) all possessed fine predictive efficacy (C-indexes: T, 0.740; N, 0.687; M, 0.823; overall survival, 0.678; progression-free survival, 0.611). In summary, we have successfully established gene expression-based models for assessing clinical characteristics of LUAD, which will assist its pathogenesis investigation and clinical intervention.

## INTRODUCTION

Around the world, lung cancer possesses the most frequent new cases and deaths among cancers [1]. Lung adenocarcinoma (LUAD) accounts for a large portion of lung cancer, urgently calling for effective treatment [2, 3].

Cancer-related genes are well-deserved targets for understanding and treating cancer, and LUAD is no exception. Indeed, almost every revolutionary breakthrough in the battle with LUAD could not run without findings of cancer-related genes [4–6]. For example, cytotoxic chemical therapies chiefly aim at genes functioning critically in basic cellular activities, and molecular targeted therapies depend principally on tumor driver genes, while immune checkpoint genes are core targets for immune therapies [6, 7]. Furthermore, tumor clinical characteristics are made up of a series of biological processes, of which underpinnings attribute to gene function [8], that is to say, cancer-related genes could also help evaluate tumor clinicopathological parameters. However, huge quantities of genes and intensive heterogeneity of LUAD both seriously curb the way to find these key players [9].

Fortunately, remarkable progress in cancer genomics and bioinformatic technology endows us with possibilities to crack such hard nut [10]. On the one hand, more well-rounded and accurate genomic profiling becomes easier to obtain [11, 12]. On the other hand, systematic and integrated analysis confers more rationality on understanding carcinogenesis, for single-gene investigation seems quite stretched facing such complex cancer pathogenesis [12, 13]. In this research, we used bioinformatic analysis to establish gene expression-based models for evaluating TNM parameters of LUAD and predicting its survival probability, which could provide insight into malignant etiology and handling methods.

## RESULTS

### Up-regulated and down-regulated DEGs were derived from LUAD transcriptome profiling

**Figure 1:**
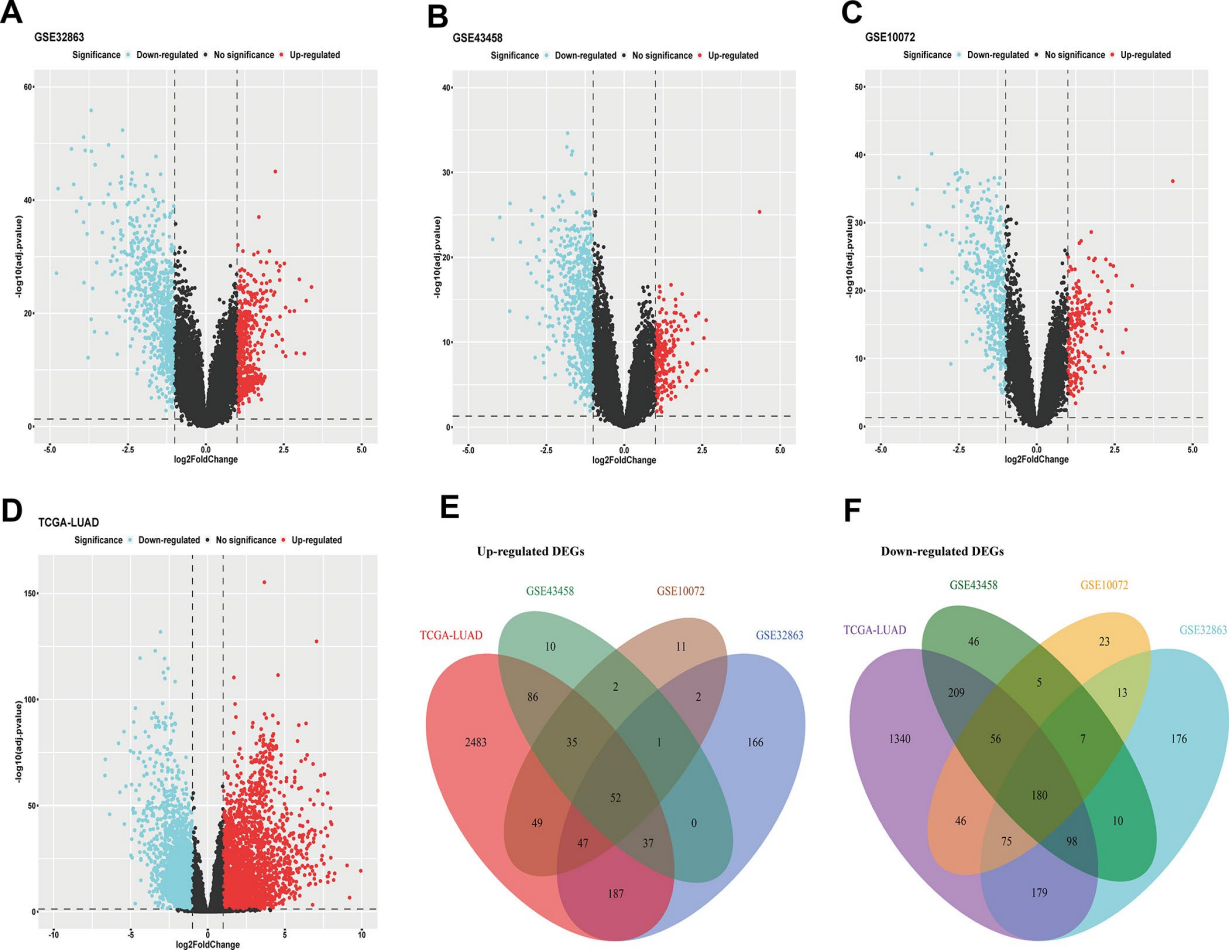
**DEGs of LUAD.** (**A**–**C**) DEGs acquired from GEO microarrays (GSE32863, GSE43458, GSE10072); (**D**) DEGs obtained from TCGA LUAD RNA-sequencing; (**E**) intersection of up-regulated DEGs; (**F**) intersection of down-regulated DEGs. DEGs, differentially expressed genes; GEO, Gene Expression Omnibus; TCGA, The Cancer Genome Atlas; LUAD, lung adenocarcinoma.

Cancer-related differentially expressed genes (DEGs) are highly correlated with tumor initiation and progression, which is quite conductive for evaluating clinical characteristics of malignancy. We acquired DEGs from three LUAD microarrays (GSE32863, GSE43458, GSE10072) using limma package (fold change >2 or fold change <0.5, Adjusted *P*-value <0.05) (Figure 1A–1C). Analogously, we used DESeq2 package to get DEGs of LUAD RNA-sequencing data from The Cancer Genome Atlas (TCGA) program (fold change >2 or fold change <0.5, Adjusted *P*-value <0.05) (Figure 1D). Then, we applied intersection analysis of these results and ultimately obtained 52 upregulated genes and 180 downregulated genes (tumor versus normal) (Figure 1E, 1F).

### Genetic annotation, biological assignment and interaction function of DEGs

**Figure 2:**
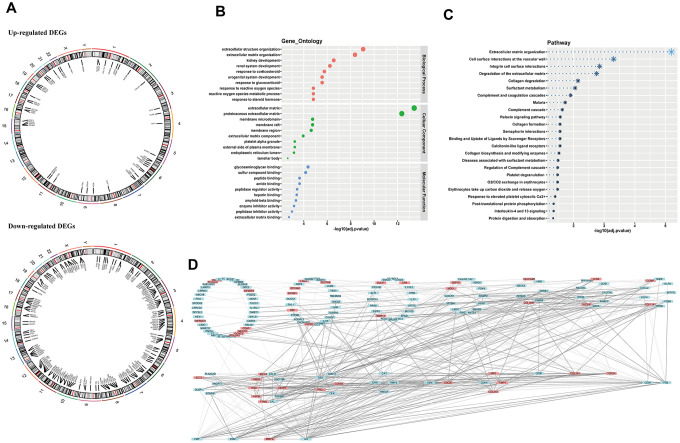
**Chromosome location, biological annotation and interaction function.** (**A**) genetic mapping of up-regulated and down-regulated DEGs; (**B**) gene ontology annotation of DEGs; (**C**) pathway enrichment of DEGs; (**D**) hierarchy of gene interaction. DEGs, differentially expressed genes.

To further comprehend these DEGs, we focused on their elementary hallmarks. First, genetic mapping atlas were drawn to show chromosome locations of these DEGs, and these DEGs had a scattered distribution in genomes (Figure 2A) (not all genes can be labeled in the figure, genomic information of the whole DEGs could be seen in Supplementary Table 1). Further, gene ontology (GO) analysis and pathway enrichment analysis both displayed significant enrichment of these DEGs in cell matrix-associated gene assemblies, which strongly indicated prominent weight of micro-environment upon carcinogenesis (Figure 2B, 2C) (Supplementary Table 2). Subsequently, we investigated interaction between DEGs in STRING database, and results suggested these DEGs had hierarchical function in malignant progression, demonstrating necessity for further filtrating (Figure 2D).

### Estimating TNM parameters of LUAD by multiple gene analysis

**Figure 3:**
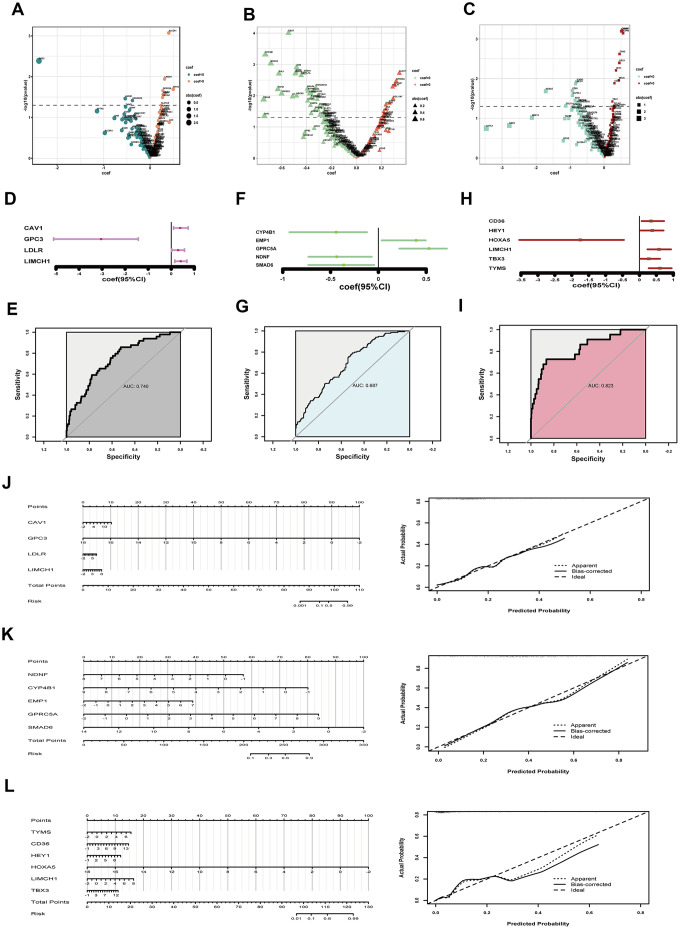
**Assessing tumor size, lymph node invasion and distant metastasis.** (**A**–**C**) coef and p value in univariate logistic regression analysis for tumor growth (**A**) lymph node invasion (**B**) and distant metastasis (**C**) respectively; (**D**) coef and 95% CI derived from the optimized model for tumor size; (**E**) ROC curve to show predictive potential of T-related model; (**F**) coef and 95% CI of the improved model for lymph node invasion; (**G**) ROC curve to exhibiting predictive efficacy of N parameter; (**H**) coef and 95% CI of the optimized model for distant metastasis; (**I**) ROC curve to exhibiting efficacy of M prediction; (**J**) Nomograph to assess T risk probability and corresponding calibration curve; (**K**) Nomograph to estimate lymph node invasion hazard and examination of efficacy; (**L**) Nomograph to assess distant metastasis risk and calibration curve showing model’s predictive potential. coef, coefficient; DEGs, differentially expressed genes; CI, confidence interval; ROC, receiver operating characteristic.

TNM staging, an assessment for tumor growth, lymph node invasion and distant metastasis, constitutes momentous facets of LUAD clinical features. We tried to unearth relationships between DEGs and TNM parameters. We first transferred TNM records to two-category dimension (T: T3-4 for higher risk indicator and T1-2 for lower indicator; N: lymph node invasion happening or not; M: having distant metastasis or not), and normalized corresponding RNA-sequencing data by Z score. Then we selected preliminarily 11 T-related DEGs, 58 N-related DEGs and 21 M-related DEGs by univariate logistic regression analysis respectively (*P*<0.05) (Figure 3A–3C). However, potent interaction among these genes indicated confounding factors existed (Supplementary Figure 1–3). So we put these primary screening genes in multivariate logistic regression models, but all showed poor significance, calling for further modification (Supplementary Figure 4–6). After step regression by Akaike information criterion (AIC), we finally built optimized models for TNM parameters. As to T parameter, we chose four genes, one gene (GPC3) functioned as a protective factor, the other three genes (CAV1, LDLR, LIMCH1) functioned as hazard factors (C-index, 0.740; R^2^, 0.173) (Figure 3D). The area under the curve (AUC) of T-related model was 0.740, indicating fine predictive effect (Figure 3E). In the improved model for lymph node invasion, we got five genes where three genes (CYP4B1, NDNF, SMAD6) showed protective efficacy, and two genes (EMP1, GPRC5A) presented risk function (C-index, 0.687; R^2^, 0.170) (Figure 3F). Similarly, the optimized N-related model possessed fine predictive potency (AUC, 0.687) (Figure 3G). For model predicting distant meta-stasis, or named hematogenous metastasis, we ultimately obtained six genes, one (HOXA5) showed protective ability, five (CD36, HEY1, LIMCH1, TBX3, TYMS) exhibited hazardous effect (Figure 3H) (C-index, 0.823; R^2^, 0.308). The optimized model of M parameter also had high consistency with reality (AUC, 0.823) (Figure 3I). Additionally, we plotted nomograph to predict risk probability of tumor growth, and calibration curve proved reasonable efficacy (Figure 3J). Analogously, lymph node invasion risk probability was presented, and the model was in good agreement with actual situation (Figure 3K). Furthermore, risk probability for distant metastasis also demonstrated fine predictive efficacy (Figure 3L).

### Establishing gene-based model for predicting overall survival probability of LUAD

**Figure 4:**
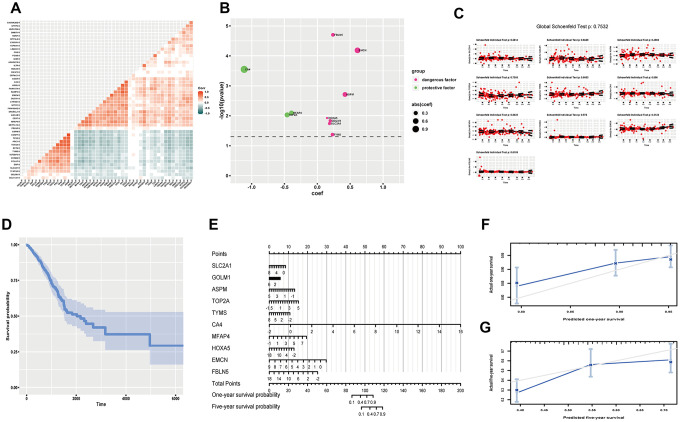
**Predicting OS probability of LUAD.** (**A**) Heatmap to exhibit correlation of genes; (**B**) coef and p value of genes in the optimized Cox proportional hazards regression model; (**C**) Residual plots to test proportional hazard assumption; (**D**) K-M curve exhibiting cumulative OS probability based on mean covariables; (**E**) Nomograph for predicting one-year and five-year OS probability; (**F**) Examining predicting efficacy of one year OS probability; (**G**) Examining predicting efficacy of five year OS probability. Corr, correlation coefficient; OS, overall survival; DEGs, differentially expressed genes; coef, coefficient; K-M, Kaplan-Meier.

Overall survival (OS) time is a momentous indicator for cancer prognosis. We tried to establish a gene expression model to predict OS probability in LUAD. We tentatively acquired 15 up-regulated genes and 34 down-regulated genes by log-rank analysis (*P*<0.05) (Supplementary Figure 7). Noticeably, univariate survival analysis of single gene is susceptible to be confounded, the correlation analysis showed signs (Figure 4A). So we applied multivariate analysis of all these genes by Cox proportional hazard regression, however, little significance reappeared (Supplementary Figure 8). We optimized the model by stepwise regression. Finally, ten genes were contained, three had protective efficacy (CA4, MFAP4, TOP2A), seven possessed hazardous potential (FBLN5, EMCN, ASPM, HOXA5, GOLM1, SLC2A1, TYMS) (C-index, 0.678; R^2^,0.149) (Figure 4B). Proportional hazards examination meets the premise condition (Figure 4C). Meanwhile, Kaplan-Meier (K-M) curve was utilized to exhibit cumulative survival probability based on means of concomitant variables (Figure 4D). Later, we used the model to predict one-year and five-year OS probability of LUAD (Figure 4E). And calibration curve proved fine predictive potency (Figure 4F, 4G).

### Reflecting progression-free survival probability of LUAD via multivariate analysis

**Figure 5:**
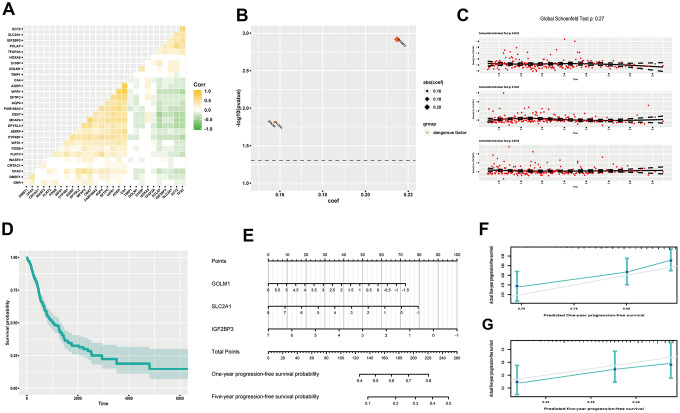
**Estimating PFS probability of LUAD.** (**A**) Heatmap of gene correlation; (**B**) coef and p value of genes in the improved Cox proportional hazards regression model; (**C**) Residual plots to examine proportional hazard hypothesis; (**D**) K-M curve exhibiting cumulative PFS probability based on average covariables; (**E**) Nomograph for estimating one-year and five-year PFS probability; (**F**) Examining predictive ability of one year PFS probability; (**G**) Examining predictive ability of five year PFS probability. Corr, correlation coefficient; PFS, progression-free survival; DEGs, differentially expressed genes; coef, coefficient; K-M, Kaplan-Meier.

As recurrence is an important indicator of worsening, progression-free survival (PFS) time functions as a crucial reference for clinical intervention. We screened out several related DEGs to reflect PFS probability of LUAD. At first, we found 9 up-regulated genes and 20 down-regulated genes by log-rank test (*P*<0.05) (Supplementary Figure 9). And confounding effect still existed (Figure 5A). We then included all these genes in a multivariate Cox proportional hazard regression model, but none genes showed significance (Supplementary Figure 10). So, optimization was done by stepwise regression. At last, we chose three genes (IGF2BP3, SLC2A1, GOLM1), which were all regarded as hazardous factors (C-index, 0.611; R^2^, 0.063) (Figure 5B). And the model abided by proportional hazards hypothesis well (Figure 5C). Further, we showed cumulative survival probability based on average scores of these genes (Figure 5D). Later, we presented nomograph to show one-year and five-year PFS probability (Figure 5E). And predictive efficacy of this model was good (Figure 5F, 5G).

## DISCUSSION

Disorder of signaling network caused by aberrant genes triggers malignant transformation and progression, which provides theoretical basis for acquiring association between genes and clinical characteristics of tumor [13, 14]. We used gene expression data to establish models reflecting tumor growth risk, metastasis hazard, and survival probability of LUAD, which will avail clinical intervention and provide insight for understanding pathogenesis.

As we all know, findings of tumor driver genes facilitate revolutionary advances in therapeutic handling of cancer. However, core genes predominating carcinogenesis are difficult to dig out. Cancer-related genes probably present different expression in cancerous tissue compared to normal tissue. The good news is that filtrating DEGs from tens thousands of genes becomes convenient, with transcriptomic and bioinformatic technology developing rapidly [12]. We first derived DEGs of LUAD from four datasets (Figure 1). Next, we investigated genetic assignment, biological enrichment and interaction function of these DEGs (Figure 2). We found these genes have not concentrated on several specific chromosomes and presented dispersive distribution. Pathway enrichments highlighted importance of microenvironments, which were tightly related to almost all malignant hallmarks of lung cancer [15]. Furthermore, result of gene interaction showed hierarchy, suggesting further digging for dominant genes was imperative.

It goes without saying that tumor growth and metastasis constitute central aspects of cancer biological characters. Tumor TNM staging remains canonical criterion for clinical evaluation and intervention [8, 16]. At present, TNM staging is mainly based on surgical exploration and imaging examination, which both could have invasive effect. However, gene expression data, acquired handily by current genomic technology, could great help to evaluate TNM parameters by appropriate statistical models. Therefore, we tried to establish correlations between TNM scores and genes by logistic regression analysis in LUAD. And optimized models showed fine potential to assess TNM parameters (Figure 3). Noticeably, survival status is regarded as an ultimate indicator for prognosis. OS and PFS both occupy crucial parts in tumor investigation and handling strategies. We utilized multivariate Cox proportional hazards regression models to predict OS and PFS based on gene expression data. Both models had reasonable predictive efficacy (Figures 4, 5).

Of course, limitations of our research still exist. First, sample size remains not adequate enough as to great heterogeneity of LUAD, which could inevitably cause confounding effect, that is why our R^2^ scores seemed not very perfect. Second, post-transcriptional regulation, post-translational modification and non-coding RNAs all contribute weightily to carcinogenesis, whereupon transcriptomic profile of only protein coding content seems not comprehensive enough to read cancer [17–19]. Third, the structure of gene-encoded products significantly affects the function of genes, which in turn affects the regulation of genes on important biological activities, such as DNA replication [20, 21], cell migration [22, 23], and etc., while nanomedicine based on molecular structure also plays an increasingly important role in cancer prevention and treatment [24–26]. Therefore, the clinical prediction based on gene expression alone, without gene structure, may not be extremely thorough and thoughtful. At last, gene expression profiling was derived from tissues, which could cause more damage compared to emerging liquid biopsies, predictive models based of which will bring much blessedness in the battle against cancer [27, 28].

In summary, we established gene expression-based models for evaluating clinical features of LUAD via integrative analysis, which will assist diagnosis and treatment of LUAD as well as enlightening investigation of cancer pathogenesis.

## MATERIALS AND METHODS

### Transcriptomic and clinical data

LUAD microarrays reflecting transcriptome profiling in tumor tissue and normal tissue were obtained from Gene Expression Omnibus (GEO) database (GSE32863, 58 tumor tissues versus 58 normal tissues; GSE43458, 80 tumor tissues versus 30 normal tissues; GSE10072, 58 tumor tissues versus 49 normal tissues) [29–31]; LUAD RNA-sequencing data and corresponding clinicopathologic annotation were derived from TCGA program, where 58 tumors and 58 normal tissues were applied for screening out DEGs, 402 samples containing availably prognostic records for survival analysis and 371 samples possessing complete TNM scorings for risk analysis respectively [32, 33].

### Genetic mapping, enrichment analysis and interaction investigation

BiomaRt, org.Hs.eg.db and RCircos packages were applied to annotate and map genetic information of genes [34–36]. And GO analysis was used to describe molecular function, cellular component, and biological process via clusterProfiler package [37]. Subsequently, clusterProfiler and ReactomePA packages were employed for pathway enrichment analysis based on Kyoto Encyclopedia of Genes and Genomes (KEGG) and Reactome database [37, 38]. Then STRING database was utilized for investigating gene interaction [39]. Adjusted *P*-value <0.05 was considered statistically significant.

### Statistical methods

DEGs derived from different transcriptome data were acquired by suitable methods respectively (Adjusted *P*-value <0.05, fold change >2 or <0.5). That is, limma package was used for microarrays, and DESeq2 package was applied for RNA-sequencing [40, 41]. Univariate and multivariate logistic regression model were used to handle two-category data. K-M curve was utilized to establish cumulative survival probability. The survival impact of single gene was estimated by log-rank test, while the Cox proportional hazards regression model was applied for multivariable analysis. Proportional hazards assumption in Cox regression was assessed by Schoenfeld residual tests. Pearson correlation analysis was used to investigate correlation. AIC was used to select and optimize models. Likelihood ratio test, Wald test, scoring test were applied for statistical hypothesis testing. *P*<0.05 was considered significant. All related arithmetic functions were practiced in R language [42].

## Supplementary Material

Supplementary Figures

Supplementary Table 1

Supplementary Table 2
