# Supplementary material for: Gene expression-based clinical predictions in lung adenocarcinoma
Source: Aging (Albany NY). 2020 Aug 5;12(15):15492–503. doi: 10.18632/aging.103721 (PMC7467359; doi:10.18632/aging.103721)
Supplement: Supplementary Figures [file aging-12-103721-s003..pdf]

SUPPLEMENTARY FIGURE

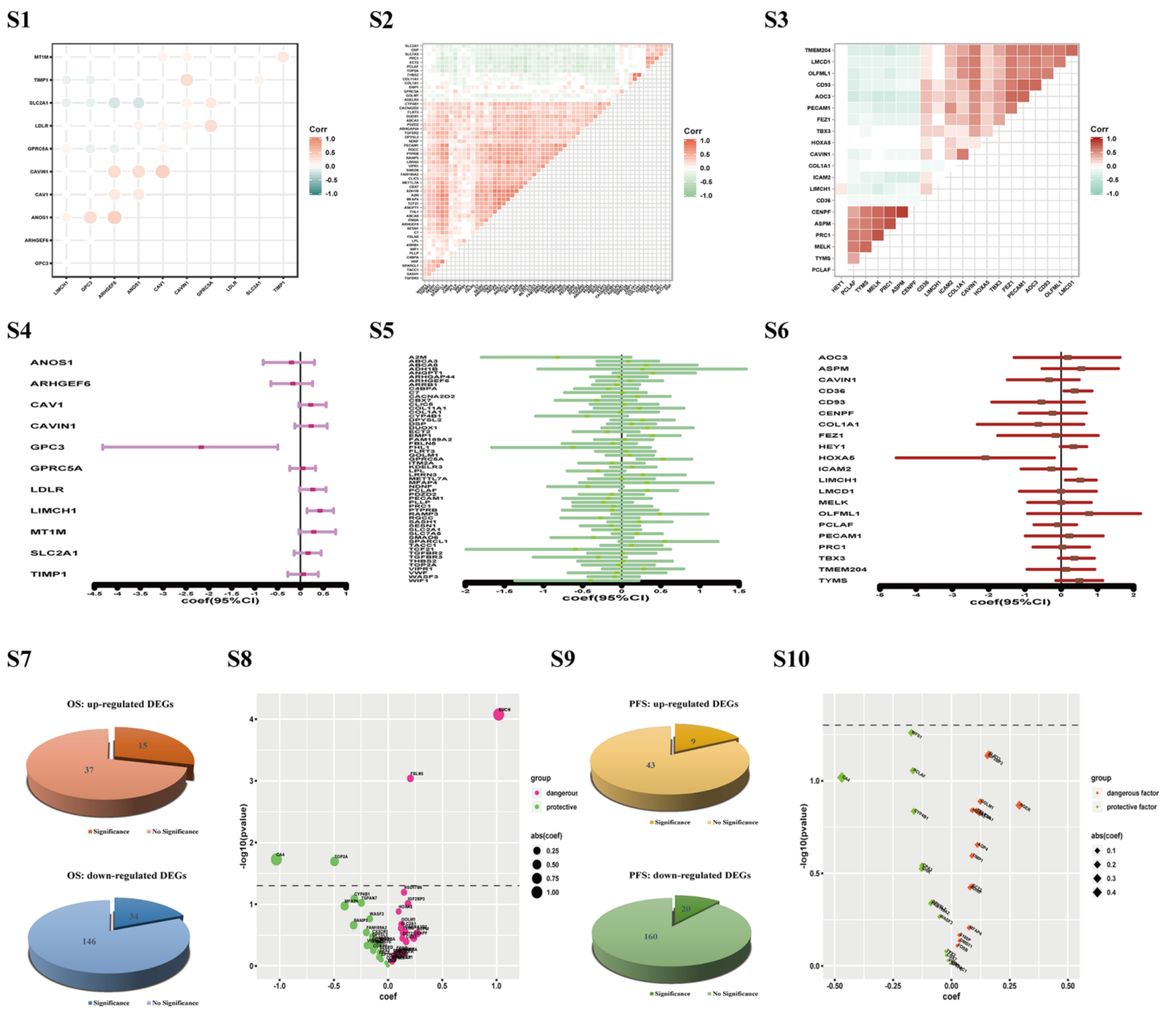

Supplementary Figure. (**S1**) (**S2**) (**S3**) correlation analysis for preliminarily filtrated DEGs for TNM parameters respectively; (**S4**) (**S5**) (**S6**) coef and 95% CI of the preliminary multivariate logistic regression models of TNM parameters respectively; (**S7**) screened OS-related DEGs by log-rank test (*P*<0.05 was considered as significance); (**S8**) coef and p value of OS-related genes in the preliminary Cox proportional hazard regression model; (**S9**) sifted PFS-related DEGs by log-rank test (*P*<0.05 was regarded as significance); (**S10**) coef and p value of PFS-related genes in the initial Cox proportional hazard regression model DEGs, differentially expressed genes; coef, coefficient; Corr, correlation coefficient; CI, confidence interval; OS, overall survival; PFS, progression-free survival.
